# Sacrificial-Rotating Rod-Based 3D Bioprinting Technique for the Development of an In Vitro Cardiovascular Model

**DOI:** 10.3390/jfb15010002

**Published:** 2023-12-19

**Authors:** Jooyoung Lee, Hyungseok Lee

**Affiliations:** 1 Department of Smart Health Science and Technology, Kangwon National University (KNU), Chuncheon 24341, Republic of Korea; weeds0555@kangwon.ac.kr; 2 Department of Mechanical and Biomedical Engineering, Kangwon National University (KNU), Chuncheon 24341, Republic of Korea

**Keywords:** extrusion-based bioprinting, GelMA, cardiovascular, sacrificial, cell alignment

## Abstract

Several studies have attempted to develop complex cardiovascular models, but the use of multiple cell types and poor cell alignments after fabrication have limited the practical application of these models. Among various bioprinting methods, extrusion-based bioprinting is the most widely used in the bioengineering field. This method not only has the potential to construct complex 3D biological structures but it also enables the alignment of cells in the printing direction owing to the application of shear stress to the cells during the printing process. Therefore, this study developed an in vitro cardiovascular model using an extrusion-based bioprinting method that utilizes a rotating rod as a printing platform. The rotating rod was made of polyvinyl alcohol (PVA) and used as a sacrificial rod. This rotating platform approach enabled the printing of longer tubular-vascular structures of multiple shapes, including disease models, and the water-soluble properties of PVA facilitated the isolation of the printed vascular models. In addition, this method enabled the printing of the endothelial cells in the bloodstream direction and smooth muscle cells in the circumferential direction to better mimic the anatomy of real blood vessels. Consequently, a cardiovascular model was successfully printed using a gelatin methacryloyl bioink with cells. In conclusion, the proposed fabrication method can facilitate the fabrication of various cardiovascular models that mimic the alignment of real blood vessels.

## 1. Introduction

Cardiovascular disease is the leading cause of mortality globally [1]. Despite breakthroughs in diagnostic technology for cardiovascular diseases, predicting the onset of dangerous cardiovascular diseases such as arteriosclerosis and aortic dissection remains a difficult task. Moreover, the basis of these diseases is closely related to turbulence [2,3], and even in vitro experiments, there are difficulties in implementing turbulence in the cardiovascular system and investigating the behavior of vascular endothelial cells owing to the difficulty in achieving the large-scale culturing of vascular endothelial cells.

Therefore, several attempts have been made to manufacture artificial blood vessels. Tissue engineering techniques [4] for fabricating vascular structures can be classified into scaffold-based processing [5,6], decellularization-based processing [7,8], and cell sheets [9,10]. The scaffold-based method is characterized with a long processing time, difficulties in controlling small-diameter vessels, and non-uniform distribution of cells in the cell seeding stage. Decellularization-based processing is characterized with difficulties in arranging cells in the desired location, and the cell sheet method is characterized with a high manufacturing cost for large blood vessels and difficulties in arranging cells in a desired position. To manufacture artificial blood vessels, the immersion of a thin rod in a hydrogel solution, to produce a tubular structure via gelation has emerged as a promising method [11]. However, despite the potential of this method for the layer-by-layer fabrication of blood vessels, it cannot be used to manufacture a complex structure and cannot imitate the unique structure of each blood vessel layer. Accordingly, efforts have been devoted to manufacture blood vessels using 3D bioprinting technology.

Among various 3D bioprinting technologies, extrusion-based bioprinting technology enables the arrangement of cells in a desired position [12]. Accordingly, various studies have attempted to develop vascular tissue structures using this method. Bioinks used in extrusion-based bioprinting must have shear thinning properties. The non-Newtonian material behavior causes the viscosity to decrease as the shear rate increases, allowing the material to flow through the nozzle in the syringe. Then, when depositing on the printing bed, the shear rate decreases and the viscosity increases rapidly to help maintain the shape of the material. To fabricate a 3D structure using an extrusion-based bioprinter, a 2D pattern is printed, followed by physical or chemical gelation, and then the 2D pattern is stacked. However, there are difficulties in maintaining the desired shape using extrusion-based bioprinting technology when a blood vessel model is manufactured by stacking in the *z*-axis. Therefore, the use of a sacrificial bath has emerged as a new method for manufacturing blood vessel models. This method involves the use of a sacrificial bath to support vascular structures during the printing process, thus enabling the vertical stacking of the vascular structures [13]. Although this method can be used to print relatively high-length vascular structures, the printed vascular structure cannot effectively mimic the spatial directions of cell alignments. Extrusion-based bioprinting technology using a coaxial nozzle has emerged as a promising method, and can print hollow vascular structures using two biomaterials at once [14]. In addition, this method can control the diameter and thickness of the vessel by changing the nozzle size and flow rate. Furthermore, it can be used to print a relatively long blood vessel. However, this method cannot be used to create disease models such as coronary arteries, patient-specific vessels, or disease models that generate turbulence, and it cannot mimic the structural features of large-diameter blood vessels [15]. Although all the three aforementioned methods can arrange cells in a desired position, they cannot be used to print a vascular structure with two layers with different cell alignment directions.

**Figure 1:**
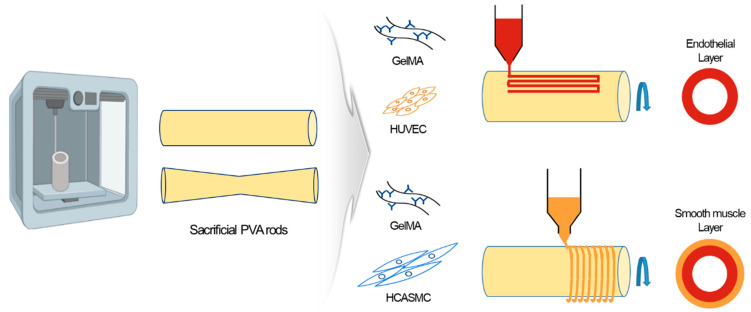
Schematic of the biofabrication process of a cardiovascular in vitro model.

Therefore, this study proposed a new biofabrication method for fabricating a vascular structure that mimics two layers of blood vessels with different cell alignment directions using a sacrificial rod (Figure 1). Using the extrusion-based bioprinting technology, cells were positioned at a desired location and cell alignment was induced using a sacrificial rod. Our proposed fabrication method can be used for disease model fabrication and can be easily used for in vitro model fabrication and diagnosis. Our novel biofabrication technique proposes a platform that can align cells in two directions in a single step and easily fabricate vascular models.

## 2. Materials and Methods

### 2.1. Gelatin–Methacryloyl (GelMA) Synthesis

First, 10 *w/v* % Type A gelatin (Type A from porcine skin 300 g Bloom, Sigma Aldrich, St. Louis, MO, USA) was added into dulbecco’s phosphate buffered saline (Gibco, Grand Island, NY, USA) at 60 °C and stirred until fully dissolved. Subsequently, 0.6 g of methacrylic anhydride (Sigma-Aldrich) was added dropwise to the gelatin solution for each gram of gelatin in the solution, and the solution was stirred at 50 °C and allowed to react for 1 h. The reaction was stopped by adding 200 mL of DPBS. Thereafter, the excess anhydride in the mixture was removed by centrifugation and decantation, after which the obtained supernatant was dialyzed against distilled water using 12–14 kDa cutoff dialysis tubing (Sigma Aldrich, D9652) for 7 days. The mixture was frozen at −80 °C overnight, and then lyophilized for 1 week.

### 2.2. Cell Preparation 

Human umbilical vein endothelial cells (HUVEC) and human coronary artery smooth muscle cells (HCASMC) were purchased from Thermo Fisher Scientific (Waltham, MA, USA). First, HUVEC was cultured in an endothelium medium (EGM-2 BulletKit, Lonza, Benicia, CA, USA) with 1% (*v*/*v*) penicillin/streptomycin (P/S, Sigma-Aldrich) at 37 °C in a humidified 5% CO_2_ atmosphere, and HCASMC was cultured in a human vascular smooth muscle cell basal medium (Medium 231, Gibco) with smooth muscle growth supplement (SMGS, Gibco) under the same conditions as the HUVEC culture.

### 2.3. Scanning Electron Microscopy (SEM) Image

To determine the effect of GelMA concentration on the pore size of the structures, samples were printed using the 3 and 5% GelMA bioinks, and then cured with UV light for 1 min. Subsequently, the samples were placed in PBS for 12 h, frozen at −80 °C for 12 h, and freeze-dried for 2 days. The lyophilized sample was coated with platinum and the pore size was measured using a scanning electron microscope (JSM-7900F, JEOL, Tokyo Japan).

### 2.4. Rheology Test

To measure the viscoelasticity of the GelMA bioink, a sample with a diameter and thickness of 25 mm and 1 mm, respectively, was prepared. Thereafter, the samples were printed using 3 and 5% GelMA and UV-cured for 1 min. Subsequently, the storage modulus and loss modulus for each GelMA sample were measured using a shear stress sweep test in the range from 0.01 to 100% at vibration frequency using an MCR 72 Rheometer (Anton Paar, Graz, Austria).

### 2.5. Swelling Test

The swelling behavior of the bioprinted structures fabricated using 3 and 5% GelMA was evaluated. The bioprinted GelMA structures (diameter and thickness of 10 mm and 2 mm, respectively) were immersed in PBS for 12 h, frozen for 12 h at −80 °C, and dried for 24 h using a lyophilizer, after which the sample was weighed (W1). Thereafter, the dried samples were immersed in PBS at 37 °C for 12 h and blotted with a KimWipe to remove the residual liquid, and the swollen weight was recorded (W2). The mass swelling ratio was measured by calculating the ratio of the hydrated weight (W2) to the dehydrated weight (W1) using the equation below:

Mass swelling ratio (%) = ((W2 − W1)/W1) × 100%.

### 2.6. PVA Degradation Test

To perform the degradation test of the PVA (eSUN, Shenzhen, China) sacrificial rod, samples with a length of 3 cm containing different infill (30 and 100%) were fabricated using a 3D printer. The prepared samples were placed in PBS solution, after which they were heated in an oven at 37 °C. The samples were taken out every 60 min and weighed, and the weight loss was calculated as a percentage.

### 2.7. Cell Encapsulation in the Hydrogels

The cardiovascular model was fabricated using the 3 and 5% GelMA bioinks. HCASMC and HUVEC were encapsulated in the 3 and 5% GelMA bioinks, respectively, at a cell concentration of 3 × 10^6^ cells/mL.

### 2.8. Cell Viability Test

The cell viability was evaluated using a Live/dead^TM^ viability/Cytotoxicity kit (L3224, Invitrogen, Carlsbad, CA, USA) when HUVEC and HCASMC were encapsulated in the GelMA bioinks and printed. The printed samples were subjected to UV irradiation for 20 s. Dead cells were stained red and live cells were stained green. Thereafter, cells were identified using a fluorescence microscope. To examine the proliferation of cells, the cells were treated with CCK-8 solution (Dojindo, Kumamoto, Japan) for 2 h, after which the absorbance of the medium was measured at 450 nm using a microplate reader (Bio Tek Instruments, Winooski, VT, USA).

### 2.9. Printing Vascular In Vitro Model 

The rods were printed using a fused deposition modeling (FDM)-based 3D printer. The rod was made of PVA filament so that it could be easily separated after printing. The printed rod was in the form of a cylinder, with a diameter of 5 mm and a length of 2 cm. The vascular model was fabricated using an extrusion-based bioprinter, and the 3% GelMA bioink was used for the endothelial layer and 5% GelMA bioink was used for the smooth muscle layer. The endothelial layer was printed to be aligned in the blood flow direction, and the smooth muscle layer was printed in the circumferential direction. A UV lamp was used to crosslink the printed structure once the smooth muscle layer printing was completed. The UV lamp was irradiated for 20 s at a 10 mm distance from the structure.

### 2.10. Cell Alignment Test

F-actin was stained green with phalloidin to confirm the alignment of the cells according to the printing direction. After printing, the cells were fixed with 4% paraformaldehyde for 15 min and permeabilized for 5 min using 0.1% triton x-100. Thereafter, blocking was performed using 2% BSA (Thermo Scientific™, Waltham, MA, USA) for 1 h, and the samples were stained with Alexa Fluor™ 488 Phalloidin (Invitrogen, Carlsbad, CA, USA) for 40 min at room temperature. Lastly, after staining with DAPI (4’,6-Diamidino-2-Phenylindole, Dihydrochloride, 10 mg, Invitrogen) for 5 min at room temperature, the staining results were confirmed using a confocal microscope.

### 2.11. Permeability Test

The barrier function of endothelial tissue was evaluated using 70 kDa FITC-conjugated dextran (Sigma Aldrich). Briefly, dextran (25 μg/mL) was added to the endothelium medium (EGM-2 BulletKit, Lonza, Benicia, CA, USA), and the printed vascular model was perfused at a rate of 20 μL/min using a syringe pump. The fluorescence intensities of the samples were detected using a fluorescence microscope.

### 2.12. Immunofluorescence Analysis

Bioprinted constructs were fixed for VE-cadherin immunofluorescence staining of endothelial cells. The cells were fixed with 4% paraformaldehyde for 15 min and permeabilized for 5 min using 0.1% triton x-100. Thereafter, blocking was performed using 2% BSA (Thermo Scientific™) for 1 h. The samples were stained with VE-cadherin polyclonal antibody (Invitrogen) for 6 h at 4 °C, then washed with PBS overnight at 4 °C, and then incubated with a Goat anti-Rabbit IgG (H + L) Superclonal Secondary Antibody, Alexa Fluor™ 488 (Invitrogen) 6 h at 4 °C. Lastly, after staining with DAPI (4’,6-Diamidino-2-Phenylindole, Dihydrochloride, 10 mg, Invitrogen) for 5 min at room temperature, the staining results were confirmed using a confocal microscope.

### 2.13. Statistical Analysis 

All variables were expressed as mean ± standard deviation (SD). An evaluation of the difference between experimental groups was performed using Student’s *t*-test, in which a *p* value of <0.05 was considered significant.

## 3. Results and Discussion

### 3.1. Evaluation of the GelMA Bioink

**Figure 2:**
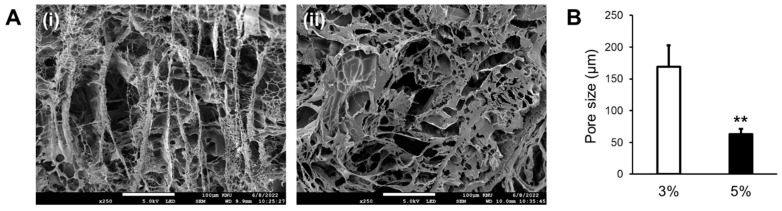
Pore size of the two different concentrations of gelatin–methacryloyl (GelMA) bioinks. (**A**) Scanning electron micrographs of (**i**) 3 and (**ii**) 5% GelMA bioinks and (**B**) pore size assessment of the two concentrations of GelMA bioinks (** *p* < 0.01).

The SEM images of the samples at each concentration are shown in Figure 2. In order for cells to grow and proliferate, the supply of oxygen and nutrients is essential, and the bioink must have porosity to supply oxygen and nutrients to the cells [16]. The average pore size of the 3 and 5% GelMA bioinks was approximately 150–200 and 50–100 μm, respectively, which was sufficient for the supply of nutrients and oxygen for cells. During the bioprinting process, different cell types were encapsulated in the different GelMA bioinks. The difference in pore size at different concentrations can help to prevent layer mixing, thereby fostering the development of more complex cardiovascular structures for in vitro model applications [17]. In addition, the concentration of the GelMA bioinks can influence the modulus of the printing materials. Therefore, the mechanical behavior of the bioinks can be controlled not only by controlling the pore size but by controlling the concentration of the GelMA bioink.

**Figure 3:**
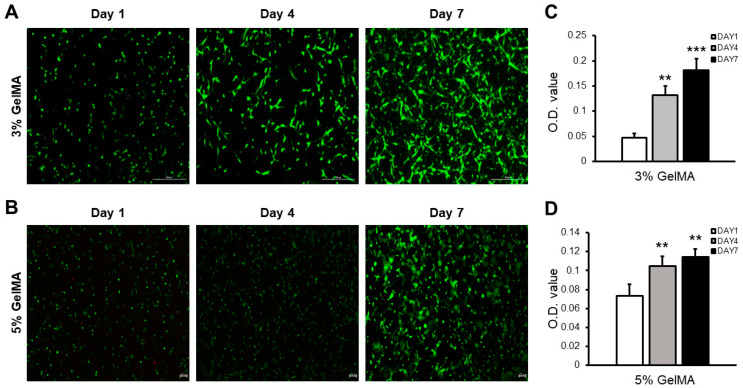
Cytotoxicity test results of GelMA. Live/dead image of cells at different GelMA concentrations: (**A**) 3 and (**B**) 5% GelMA bioinks (scale bar 500 and 100 μm, respectively). Cell proliferation with a change in the GelMA concentrations: (**C**) 3% and (**D**) 5% GelMA bioinks (** *p* < 0.01, *** *p* < 0.001).

As mentioned previously, the pore size of the bioink decreased with an increase in the concentration of the GelMA bioink, which can affect the cell viability and proliferation [18]. To evaluate the cytotoxicity and effect of the pore size, cells with two different GelMA bioinks were printed by encapsulating the cells in GelMA bioinks, followed by a bioprinting process. Printed samples were cured by UV irradiation for 20 s [19]. As shown in Figure 3A,B, the cells were alive in both the 3 and 5% GelMA bioinks, and the number of viable cells increased with an increase in the cell culture period. 

These results indicate that the bioprinting of the cells from the bioink preparation stage to the printing process did not induce any toxicity. And the results indicate that UV irradiation for 20 s does not induce significant cell death. Furthermore, the examination of the O.D. value of the cells during the culture period revealed that the 3% GelMA bioink exhibited higher O.D. values compared to the 5% GelMA bioink. Although both GelMA bioinks contained similar number of cells after the bioprinting process, cells in 3% GelMA bioink exhibited significantly higher potential for cell proliferation owing to its higher pore size value (Figure 2) and porosity. Although the 3% GelMA bioink exhibited a higher cell proliferation rate, printing possibilities and bioink modulus have to be considered when selecting the bioprinting strategy.

### 3.2. Rheological Properties of GelMA Bioinks

The viscoelasticity was measured for two main reasons [20]: first, to examine the strength of the material, and second, to examine whether the material can be used in the bioprinting process. For the material to be usable in the bioprinting process, the bioink must have shape retention and gelation properties after printing. For the printed structures to maintain their shape, bioinks to be used in extrusion based bioprinting must have shear thinning properties. Shear stress can be applied to the bioink to be printed in a desired shape, and the moment the shear stress decreases after printing, the bioink maintains the desired shape [21]. Gelation of the bioink may be performed by a physical method such as temperature control or a chemical method such as UV irradiation. In the case of GelMA, since it is a gelatin-based photocurable hydrogel, the degree of gelation can be controlled by temperature and irradiating UV light after printing.

**Figure 4:**
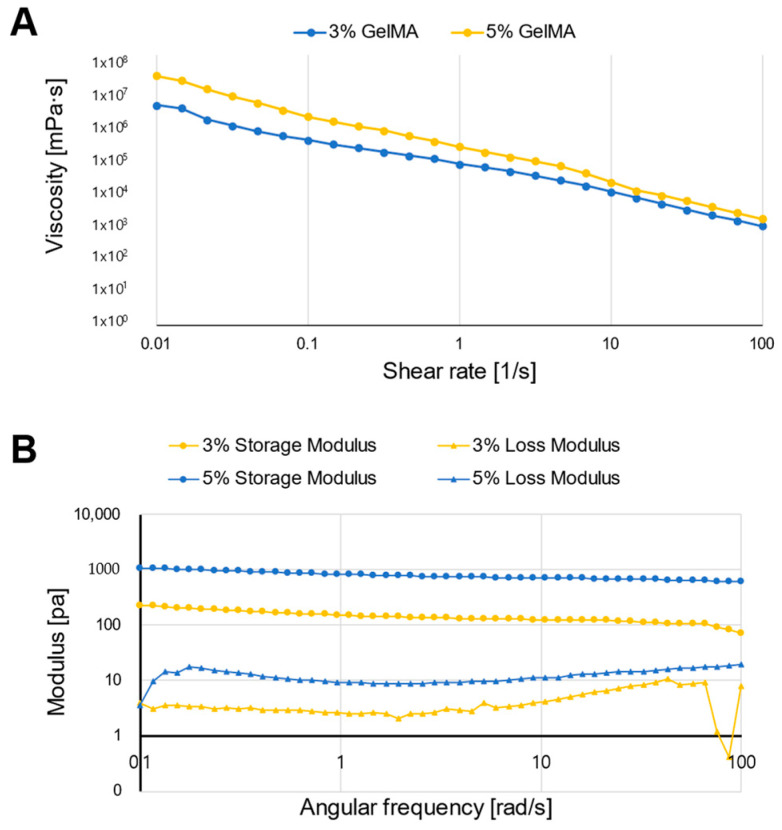
Parameterization of the two GelMA bioinks. (**A**) Viscosity of the 3 and 5% GelMA bioinks, (**B**) rheological assessment of the 3 and 5% GelMA bioinks.

The viscosity measurement results confirmed the shear thinning properties of the GelMA bioinks, indicating that the 3 and 5% GelMA bioinks can be used for extrusion-based bioprinting (Figure 4A). The viscoelasticity measurement results of the two GelMA bioinks are shown in Figure 4B. The results confirmed that the storage modulus of the two GelMA bioinks was larger than their loss modulus. The storage modulus measures the stored energy of the printed structure and represents the elastic component, and the loss modulus measures the released energy of the printed structure and represents the viscous component [22]. If the storage modulus is greater than the loss modulus, the shape can be maintained after printing, and the rheological test results indicate that the printed blood vessel model can maintain its tubular shape. The minimum pore size required for printing is influenced by various factors, such as cell type and function, with pore sizes larger than 30 μm required to allow nutrient diffusion into the scaffold via blood vessels [23,24]. The pore size is determined by the hydrogel concentration, which has an inverse relationship: higher concentrations correspond to smaller pore sizes and higher modulus. Furthermore, as smooth muscle cell layers possess higher mechanical properties than endothelial cell layers, the higher-concentration GelMA bioink was used as the smooth muscle cell layer of the cardiovascular model.

### 3.3. Evaluation of the PVA Sacrificial Rod as a Bioprinting Platform

PVA is a water soluble, biocompatible, and synthetic polymer that can be used as a sacrificial material during the 3D printing process [25]. However, there have been less attempts using PVA for bioprinting cellular components to construct 3D complex biological structures. Therefore, in this study, we employed a PVA polymer as a sacrificial material (rotating rod) to fabricate the cardiovascular in vitro model. The degree of decomposition of the PVA with a change in the filling of the PVA rotating rod used as the sacrificial rod was evaluated. 

**Figure 5:**
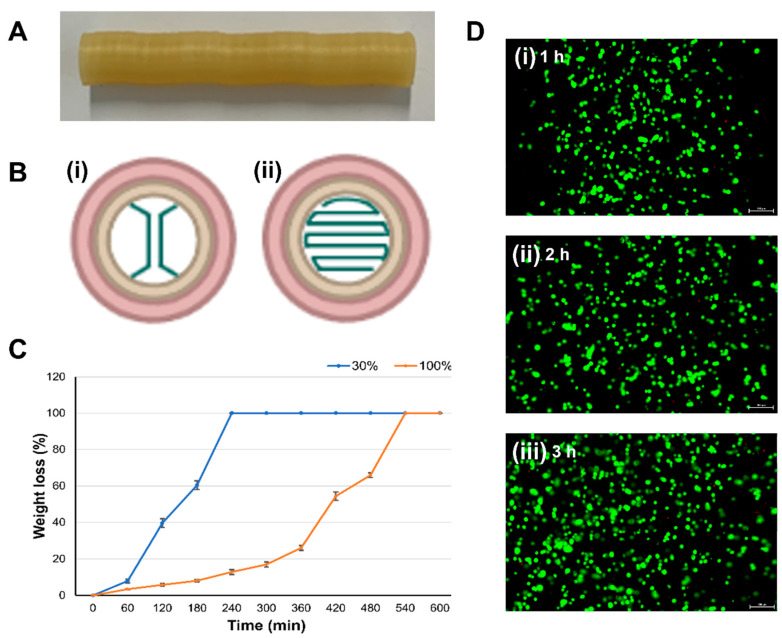
(**A**) Gross image of the sacrificial PVA rod, (**B**) infill patterns of the 30 and 100% of PVA sacrificial rod (**i**. 30%, **ii**. 100%), (**C**) result of the PVA sacrificial rod degradation test, (**D**) cytotoxicity test results according to PVA degradation.

The PVA degradation test results of infill 30% and 100% samples are shown in Figure 5. The 30% and 100% infill samples were completely decomposed after 240 and 540 min, respectively. In addition, the 30% infill sample could not maintain its shape after approximately 120 min. In contrast, the 100% infilled PVA sacrificial rod maintained its shape for a very long time. The 30% infill sample decomposed completely in PBS within 3 h, effectively fulfilling its role as a sacrificial material [26,27]. However, the 100% infill sample retained its shape for an extended period and resisted easy separation from the blood vessel model. While printing GelMA bioinks as extracellular matrix materials for cardiovascular model, we cross-linked the bioinks right after the printing process. Therefore, a sacrificial rod with a faster degradation time will be more suitable for the structure and biological culture. For the fast separation of the printed blood vessel model and the sacrificial rod, the infill of the PVA sacrificial rod was set at 30%. It takes up to 3 h to separate the printed blood vessel model and PVA. During the decomposition of PVA in the medium, it was confirmed whether the PVA solution causes side effects on cell viability. Images evaluating cell viability are shown in Figure 5D. PVA is a synthetic polymer that can potentially be used in vascular grafts [28]. Furthermore, the cells were alive during the 3 h that PVA was degraded. This means that PVA has no effect on cells while it is degraded and separated from the vascular model.

### 3.4. Cell Alignment by the Extrusion Printing Process

**Figure 6:**
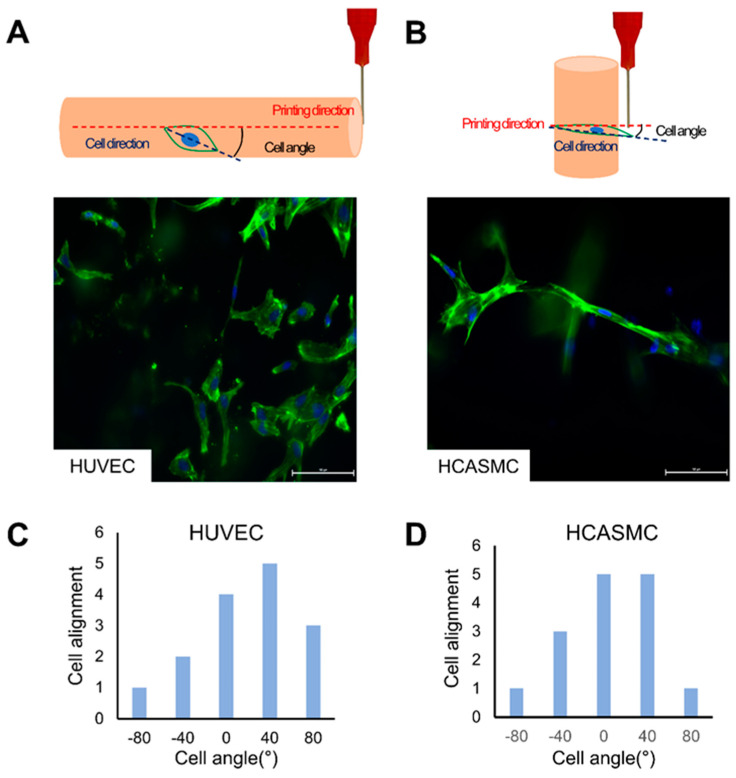
Cell alignment according to the printing direction. Image of cell alignment according to the dapi/phalloidin staining of (**A**) HUVECs and (**B**) HCASMCs (scale bar: 100 µm), (**C**,**D**) graphs showing the alignment of cells quantitatively by measuring the cell angle of F-actin.

When using an extrusion-based bioprinter, the cells are subjected to shear stress by the pressure applied to the cells [29]. If the shear stress applied to the cells is too high, the cells will die, but a moderate shear stress will enable the alignment of the cells in the printing direction [30]. Figure 6A,B shows the results of F-actin staining. To quantitatively observe the direction of cells, the alignment distribution of F-actin was measured using a phalloidin image. Both HUVECs and HCASMCs were aligned in the range of −40° to 40°. Here, the 3 and 5% GelMA bioinks were used for HCASMC and HUVEC, respectively. When a higher-concentration bioink was used, a more aligned cellular behavior was observed, which may be attributed to the increased difficulties in cell movement at a higher GelMA bioink concentration.

### 3.5. Bilayer Blood Vessel-like Construct Printed Using 3D Bioprinting Technology

Cells encapsulated in GelMA bioinks were printed on the pre-prepared PVA sacrificial rods. After the tubular structure was stably printed on the PVA rod and cured by UV radiation, the water-soluble property of the PVA filament was utilized to melt the PVA rod in the medium to produce a cardiovascular in vitro model. As described previously, by selecting an appropriate infill pattern of the PVA sacrificial rod, the use of the 30% infill sacrificial rod facilitated the separation of the printed vascular structure from the rod. The blood vessel endothelial layer was printed in the direction of blood flow, and the smooth muscle layer was printed in the circumferential direction according to the cell alignment direction in an actual blood vessel.

**Figure 7:**
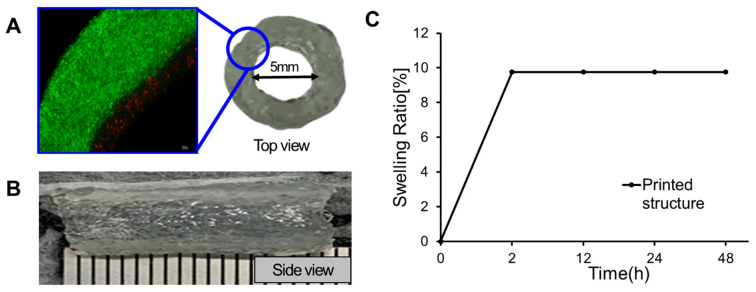
Printed bilayer blood vessel-like construct. (**A**) Bio-layered cell-printed cardiovascular structure. (**B**) Side view (gross image) of the bioprinted vascular structure, and (**C**) swelling test result of the printed in-vitro cardiovascular model.

Figure 7A,B shows the printed blood vessel model. Before printing the model, HUVEC was stained red and HCASMC was stained green. In actual blood vessels, the endothelial cell layer consists of a single layer of endothelial cells and is thinner than the smooth muscle layer. By adjusting the printing pressure and the moving speed of the nozzle, the endothelial layer was printed thinner like the structure of an actual blood vessel, and it can be confirmed that the printing was similar to the actual blood vessel through the stained image. As two different GelMA bioinks with different pore sizes and concentrations were utilized, the vascular structure could be printed by separating it into two (endothelium and smooth muscle) layers. While studies are ongoing to develop multi-scale native-mimicking vascular 3D models using various hybrid techniques, cell alignment has proven challenging. However, our method enables the alignment of two types of cells in opposite directions, closely mimicking the cellular arrangement observed in actual blood vessels [31,32,33]. Furthermore, compared to other vascular structure fabrication methods, this method could be used to fabricate large diameter-sized vascular structure (diameter over than 5 mm) in a stable manner. The swelling characteristic of the printed structure is critical for tissue engineering applications as they directly influence solute mobility and diffusion, as well as the mechanical properties of the scaffold [34]. The swelling properties of the printed structure were observed to be constant from 2 h after printing, indicating that the printed structure maintained its shape 2 h after printing. 

### 3.6. Fabrication of Custom Structures through 3D Bioprinting

**Figure 8:**
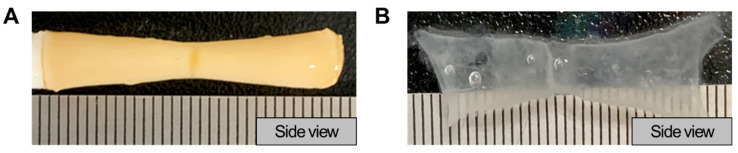
Vessel-like structure in printed stenotic models. (**A**) Image before separation with PVA rod. (**B**) Image after separation with PVA rod.

As various types of PVA sacrificial rods can be manufactured using 3D printing technology, disease models for each patient and turbulent disease models can be printed. The image of the endothelial cell layer printed on a PVA rod simulating the area with stenosis is shown in Figure 8. The turbulent disease model mimics coronary artery stenosis, in which narrowing of the tubular structure occurs. The printing of the disease model in which turbulence occurs mimics the formation of turbulence as the flow rate increases by fabricating the part where the stenosis occurs in the tubular structure. When the stenosis rate is 50% or above, a negative pressure is observed in the downstream region of the stenosis, and the value increases as the stenosis rate increases [35]. Therefore, the model in which stenosis occurred produced a PVA rod with an inner diameter reduced by 50% based on the average diameter of the coronary artery. If we can measure the pressure of the stenosis in the vascular model we designed, it would provide a numerical means to ascertain the potential generation of turbulence. This capability will enable us to investigate diseases caused by turbulence. As the PVA rod can be manufactured in various ways, it is possible to manufacture a patient-specific stenosis model. Therefore, by preparing a patient-specific stenosis model, there is a high possibility of patient-specific disease treatment using the proposed bioprinting method. 

Moreover, GelMA is a substance used as a bioink and is characterized by slow degradation, making it suitable for in vivo blood vessel formation. Consequently, the bioprinted vascular model has the potential to be a viable candidate for use as a vascular graft [36,37]. The ability to control the drug release profile exists by modifying the pore size according to GelMA concentration [38,39]. Consequently, drug delivery can be controlled by varying the concentration of GelMA in both the endothelial and smooth muscle layers within the vascular system. This implies that the fabricated vascular structure can be used as a drug delivery system. Nonetheless, the fabrication of branched blood vessels using bioprinting techniques including cells poses significant challenges. Consequently, there is a compelling imperative for additional research efforts to facilitate the successful printing of branched components

### 3.7. Tissue Maturation/Maintained Layer Specificity of HUVECs and SMCs In Vitro

**Figure 9:**
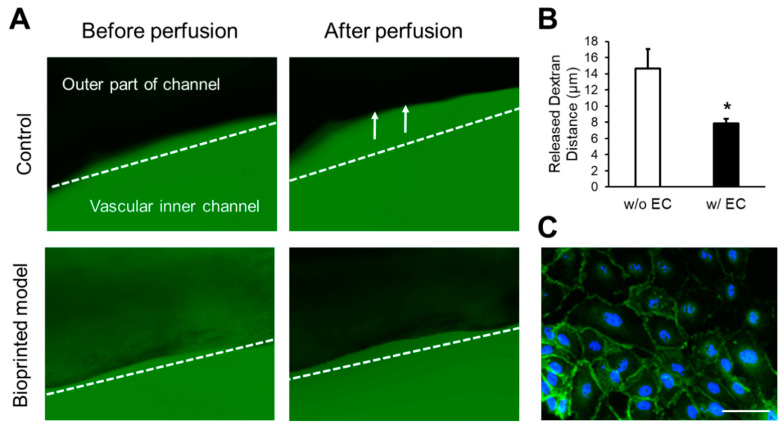
Identification of tissue maturation. (**A**) Images taken under a microscope after dextran diffusion, (**B**) graph quantifying the image (* *p* < 0.001), (**C**) immunofluorescent staining of the tubular construct at day 7 (scale bar: 100 µm).

The selective permeability of the printed structure was investigated to evaluate the function of the cardiovascular in vitro model. Figure 9A,B shows the permeability test results. The comparison of a vascular model printed without cells and the vascular model printed by encapsulating cells revealed that after 30 min of the dextran diffusion test, the presence of cells in the structure (i.e., the structure printed using the newly proposed method) reduced the diffusion of dextran. The image shown in Figure 9C, obtained after 7 days of culture and stained using immunofluorescence, confirms the presence of VE-cadherin expression. VE-cadherin is a marker that indicates the presence of junctions between endothelial cells, and its satisfactory expression suggests that the junctions between endothelial cells are well-formed and functional. This indicates that the proposed method of printing and preparing the endothelial layer is effective in mimicking the functions of cardiovascular tissues, and can be considered successful.

## 4. Conclusions

This study demonstrated the possibility of fabricating cardiovascular models using PVA rods, and fabricating cardiovascular models that induce cell alignment using extrusion-based bioprinting technology. The results revealed that the difference in the pore size of GelMA prevented cell migration. In addition, the cell encapsulated in GelMA bioink survived for up to 7 days of culture, and the expression of cell-specific biomarkers revealed that GelMA bioink provided a microenvironment that supports cell growth. As various disease models can be manufactured, the proposed method will enable the fabrication of patient-specific vascular models. As a future research direction, experiments on diagnostic factors that cause diseases will be conducted through integration with the culture system.

## Data Availability

The data presented in this study are available on request from the corresponding author.
